# Public Acceptance of and Willingness to Pay for Mosquito Control, Texas, USA

**DOI:** 10.3201/eid2802.210501

**Published:** 2022-02

**Authors:** Katherine L. Dickinson, Natalie Banacos, Ester Carbajal, Nina Dacko, Chris Fredregill, Steven Hinojosa, Jose G. Juarez, Caroline Weldon, Gabriel L. Hamer

**Affiliations:** University of Colorado Anschutz, Aurora, Colorado, USA (K.L. Dickinson);; Boston University School of Public Health, Boston, Massachusetts, USA (N. Banacos);; Texas A&M University, College Station, Texas, USA (E. Carbajal, J.G. Juarez, G.L. Hamer);; Tarrant County Health Department, Fort Worth, Texas, USA (N. Dacko);; Harris County Public Health, Houston, Texas, USA (C. Fredregill);; Hidalgo County Health and Human Services, Edinburg, Texas, USA (S. Hinojosa);; University of Texas Medical Branch, Galveston, Texas, USA (C. Weldon)

**Keywords:** vector-borne infections, meningitis/encephalitis, mosquito-borne, mosquito control, questionnaire, public opinion, viruses, Texas, United States, *Suggested citation for this article*: Dickinson KL, Banacos N, Carbajal E, Dacko N, Fredregill C, Hinojosa S, et al. Public acceptance of and willingness to pay for mosquito control, Texas, USA. Emerg Infect Dis. 2022 Feb [*date cited*]. https://doi.org/10.3201/eid2802.210501

## Abstract

Mosquito control is essential to reduce vectorborne disease risk. We surveyed residents in Harris, Tarrant, and Hidalgo Counties, Texas, USA, to estimate willingness-to-pay for mosquito control and acceptance of control methods. Results show an unmet demand for expanded mosquito control that could be funded through local taxes or fees.

Public health responses are not purely technical undertakings; these responses happen within and are affected by their social and economic contexts. Whether or not these efforts succeed depends on public acceptance and response and on financial viability (*1*). To fully assess which vectorborne disease control methods will be sustainable and effective, public health practitioners and researchers must understand public perceptions and acceptance of different approaches.

Vector control is a particularly salient public health topic in Texas. The state had one of the highest rates of West Nile virus (WNV) in 2002–2019 (*2*); Texas and Florida are the 2 US states with periodic local transmission of *Aedes* spp. mosquito–borne viruses such as dengue virus (DENV), Zika virus (ZIKV), and chikungunya virus (CHIKV) (*3*). Although Texas shares a border with Mexico, which has had outbreaks of these 3 viruses, and despite the substantial impact of mosquitoborne disease on public health across the state, very few of its cities or counties have organized vector control programs. Those that do focus primarily on nuisance mosquitoes, and disease-carrying mosquitoes are usually targeted in response to cases rather than preventively (*4*). State law requires a petition and a vote to create a new mosquito control district, but establishing such districts requires raising taxes, which is rarely popular among the Texas electorate (*5*).

The objective of this study was to determine public attitudes toward and willingness to pay for mosquito control in Harris, Tarrant, and Hidalgo Counties, regions with varying risk for mosquitoborne pathogens, socioeconomic conditions, and current mosquito control practices (Appendix 1 Figure 1). Participants provided written consent to take the survey. The Colorado Multiple Institutional Review Board (COMIRB) approved the study on March 2, 2018 (protocol no. 18-0348), and the Texas A&M University Institutional Review Board approved the study on July 2, 2018, after determining the proposed activity was not research involving human subjects (protocol no. 2018-0774). 

## The Study

We conducted a public survey (Appendix 2) to answer 2 research questions: 1) How much are residents willing to pay for increased mosquito control, and how does willingness to pay vary across counties and with individual characteristics?; 2) To what extent do residents support or oppose different methods for controlling mosquitoes, and how does level of support vary across counties and with individual characteristics?

To measure willingness to pay, we used a triple-bounded dichotomous choice contingent valuation question design (*6*). We presented participants with background information about current mosquito control methods in their county, including the annual budget per person. We then asked whether they would support a proposal to expand mosquito control efforts in their county at different annual fees; their answers enabled us to estimate a WTP range for each respondent.

We then presented participants with fact sheets on 6 mosquito control methods: adulticides, larvicides, traps, and mass releases of genetically modified mosquitoes, sterile male mosquitoes, or mosquitoes artificially infected with *Wolbachia* bacteria. After viewing information about the control methods, participants were asked to indicate their level of support or opposition to the use of each method as part of an expanded mosquito control program in their area; responses were strongly oppose, oppose, neutral/no opinion, support, strongly support.

In total, 1,831 Texas residents participated in this survey: 610 from Harris County, 609 from Tarrant County, and 612 from Hidalgo County (Appendix 1 Table 1). Participants were willing to pay $53.15 (95% CI $50.09–$56.21) per year on average to expand mosquito control in their area. Harris County residents expressed the highest WTP values at an average of $56.74 (95% CI $50.91–$62.57), followed by Hidalgo County residents at $51.87 (95% CI $46.60–$57.14) and Tarrant County residents at $51.74 (95% CI $46.72–$56.76). Differences in WTP values across counties were not statistically significant (χ^2^ = 1.22; p = 0.54).

**Figure 1:**
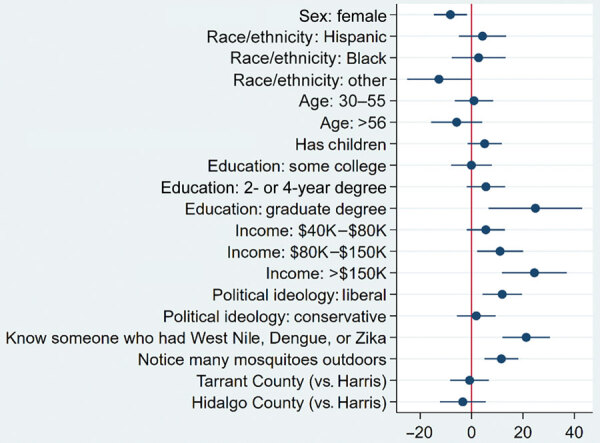
Interval censored regression results showing variation in public willingness to pay for vector control as a function of individual characteristics and county, Harris, Tarrant, and Hidalgo Counties, Texas, USA. Dots represent point estimates and bars 95% CIs. Red line represents the reference category (e.g., male sex, non-Hispanic White race/ethnicity, respondents <30 years of age, respondents without children) (Appendix 1 Table 1).

Women were willing to pay $9 less for vector control than men (Figure 1). Persons with graduate degrees were willing to pay $25 more than those with a high school or lower education level, and participants were willing to pay more with increasing income (controlling for education). Participants who identified as politically liberal were willing to pay about $12 more than those who identified as moderate. On average, persons who reported knowing someone who had had WNV, DENV, or ZIKV were willing to pay $21 more than those who did not, and persons who noticed many mosquitoes outdoors at the time of the survey were willing to pay $12 more than those who did not (Figure 1).

**Figure 2:**
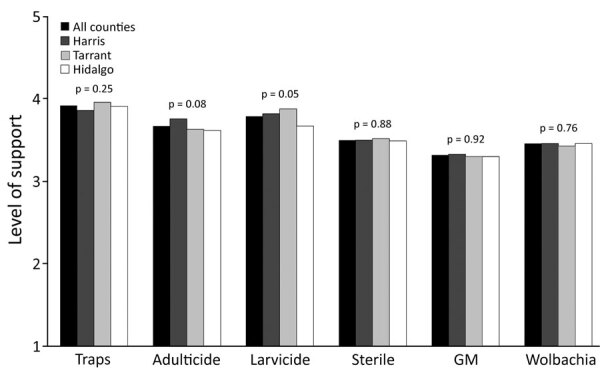
Average (mean) level of public support for mosquito control methods by county, Harris, Tarrant, and Hidalgo Counties, Texas, USA. Level 1, strongly oppose; 2, oppose; 3, neutral; 4, support; 5, strongly support. Kruskal-Wallis test used for differences in level of support across counties. GM, genetic modification.

Levels of support for the 6 different control methods were similar across counties (Figure 2). Lethal traps were the most favorable mosquito control method. Releasing genetically modified (GM) mosquitoes was the least favorable approach, although most participants still supported it. Support for different control methods varied with individual characteristics (Appendix 1 Figure 2). Women were less supportive of the 3 modified mosquito control methods (GM mosquitoes, sterile males, and *Wolbachia* infected) than men. Compared with White respondents, Black respondents were less supportive of the sterile-male method. Respondents >30 years of age tended to be more supportive of several control methods than younger respondents. Higher education was somewhat predictive of support for adulticides, larvicides, and the sterile male method; respondents in the highest income group were more supportive of traps, adulticides, and larvicides. Respondents who identified as politically conservative were more supportive of adulticides compared with the politically moderate, whereas liberal respondents were somewhat more supportive of GM mosquitoes. Support for adulticides and the *Wolbachia* and GM approaches was also higher among respondents who knew someone who had had WNV, DENV, or ZIKV; respondents who reported noticing many mosquitoes outdoors were more supportive of adulticides and larvicides. Compared with Harris County respondents, Tarrant County participants were more supportive of traps and less supportive of adulticides.

When asked an open-ended question about why they supported or opposed different control methods, many participants said they were in favor of anything that would eliminate mosquitoes, to get rid of the nuisance or protect their families and communities from disease. Others emphasized that they would prefer a control method that was proven safe for humans and other animals. Whereas some expressed skepticism about the safety of GM mosquito options, others simply did not want more mosquitoes released in their area. “Oppose anything with genetically modified anything,” wrote one participant. “That’s how *Jurassic Park* began.” In contrast, a participant who was in favor of the GM methods responded, “… I love the idea of using mosquitoes to fight mosquitoes.”

## Conclusions

Measuring public demand and support for mosquito control is crucial to successful vectorborne disease prevention strategy. Our results show a demand for expanded mosquito control that could be met through programs funded with local taxes or fees. Follow-up work should assess the feasibility of establishing such programs, examining policies that could enable or prevent local programs from emerging. Community engagement can promote mutual understanding and guide sustainable public health strategies to address the threat of vectorborne disease.

Appendix 1 Additional information from the study of willingness to pay for and acceptance of mosquito control, Texas, USA.

Appendix 2 Survey instrument and fact sheets used in study of willingness to pay for and acceptance of mosquito control, Texas, USA.
