# Supplementary material for: Public Acceptance of and Willingness to Pay for Mosquito Control, Texas, USA
Source: Emerg Infect Dis. 2022 Feb;28(2):425–8. doi: 10.3201/eid2802.210501 (PMC8798704; doi:10.3201/eid2802.210501)
Supplement: Appendix 1 — Additional information from the study of willingness to pay for and acceptance of mosquito control, Texas, USA. [file 21-0501-Techapp-s1.pdf]

Article DOI: https://doi.org/10.3201/eid2801.210501

Public Acceptance of and Willingness to Pay for Mosquito Control, Texas, USA

Appendix 1

A lack of community, political, or bureaucratic support can hinder effective mosquito control efforts. For example, the 2012 West Nile virus (WNV) outbreak in Dallas County, TX, resulted in 398 WNV-related illnesses and 19 deaths. Aerial adulticide applications are uncommon in Dallas and it had been 45 years since the last aerial spray event (*1*,*2*). Accordingly, the approval process to use aerial adulticides amid the epidemic was extensive and spraying was delayed until Aug. 16, when rates of *Culex* spp. mosquito infection with WNV and human West Nile neuroinvasive disease (WNND) were already declining (*3*). Furthermore, 13 of 44 municipalities opted out of aerial spraying (*4*), due in part to public opposition to control efforts (*5*). In total, aerial spraying cost about $1.6 million, well below the estimated WNV morbidity-related costs of $274.3 million across Texas (*6*), with Dallas County and surrounding counties leading the nation in the number of cases (*7*). Clearer criteria for initiating emergency vector control practices, along with sustained public education and support, may have averted many cases and deaths.

Local representatives and taxpayer-funded vector control authorities may have different perceptions of mosquito control than the publics they serve. Following a 2009–2010 dengue outbreak in Florida, Key West officials sought to launch a trial of genetically modified (GM) mosquitoes that would reduce wild populations by producing offspring that died before reaching maturity. While vocal opponents organized in opposition to this approach, a 2012 survey of Key West residents showed that the majority were supportive of this idea (*8*). However, a subsequent survey in 2015 showed that most residents did not support this proposition (*9*). In 2016, residents of Key Haven, where a trial was proposed, rejected a ballot measure to move forward with this trial, while another ballot measure open to all surrounding Monroe County residents passed (*10*). In August 2020, despite vocal public opposition, the Florida Keys Mosquito Control Board voted to initiate a trial release of GM mosquitoes in 2021 – the strategy for executing this trial and addressing community concerns remains to be seen (*11*). To overcome opposition to potentially

effective mosquito control methods, a better understanding of what motivates public opinion is needed. In the Key West example, survey results showed that people opposed to the GM mosquito trials tended to cite concerns about disturbing ecosystems and using unproven technologies (*8*,*9*) whereas some who supported the trial said they thought this method was more natural and less harmful than chemical-based controls (*8*). Perceptions of the risks that mosquitoes pose, and their general nuisance level, can also be important motivators of support for control efforts. A survey of Madison, Wisconsin residents showed that dislike for nuisance mosquitoes was a stronger driver of willingness-to-pay for mosquito control than concerns about disease (*12*). Concerns about the environmental impacts of mosquito control were found to decrease willingness to pay for mosquito control in Key West, Florida (*13*). Recent studies have also found that residents would be willingness to pay more to expand mosquito control programs in New Jersey (*14*) and North Carolina (*15*).

This study protocol was reviewed and approved by the Colorado Multiple Institutional Review Board (COMIRB) (Protocol #: 18-0348, approved March 2, 2018) and the Texas A&M University Institutional Review Board determined the proposed activity was not research involving human subjects on July 2, 2018 (Protocol #: 2018-0774). Participants provided written consent to take the survey.

Methods

Survey Design and Administration

To inform the design of our survey, we interviewed key stakeholders involved in mosquito control at state level and in Harris, Tarrant, and Hidalgo counties in Texas (Appendix 1 Figure 1).

Using findings from these interviews, we developed a survey to address the following: 1) household characteristics, 2) perceived impacts of mosquitoes on quality of life, 3) vectorborne disease knowledge and concerns, 4) mosquito avoidance and control behaviors, 5) willingness-to-pay for expanded mosquito control, and 6) opinions on specific control methods (e.g., adulticides, traps). The full survey is available below. After a pilot study to recruit by mail that resulted in a low response rate (~10%), we contracted Qualtrics, a software and analytics

company, to recruit survey participants. Qualtrics uses multiple actively managed panels of survey participants recruited for academic and market research. While relying on Qualtrics panels limited our ability to recruit at random from specific geographic areas within each county, it did allow us to set quotas for gender, race/ethnicity, and median household income to obtain samples that largely reflect the demographic makeup of each county. The survey was administered to a total of 1,831 persons during October 24–November 15, 2019. Participants could choose to take the survey in English or Spanish.

Measuring Willingness to Pay

Appendix 1 Figure 2 depicts the design of our triple-bounded WTP question format. A key benefit of this approach over alternative dichotomous choice contingent valuation question formats is that it gathers more information from each respondent, allowing us to estimate WTP more precisely for a given sample size (*16*). Our approach is largely consistent with recommended best practices in WTP measurement (e.g., the NOAA Blue Ribbon panel report (*17*)). For example, we use a referendum format (yes or no vote on a proposed ballot measure), allow a "don't know" option, emphasize that all answers are reasonable and valid, and remind the respondent that money used toward mosquito control would not be available for other uses. A sample question is below:

> Suppose that there were a proposal on the next election ballot to expand mosquito control across the county. If the proposal passes, the number of mosquitoes in this area would be cut in half. To fund this expansion, your household and others in the county at your income level would be charged a fee of $XX once per year. Would you support this proposal?

The fee amount for this initial question was randomized across respondents ($5, $10, $25, $50, or $100). Participants who said yes to the initial fee were then asked if they would support a fee of twice that amount. If they said yes to that, the fee doubled again. Participants who said no to the initial fee were asked if they would support a fee that was half the initial amount. If they said no again, the fee was halved again (Appendix 1 Figure 2).

Resulting data include upper and lower bounds on WTP for each participant. The exceptions are for participants that answered "Yes" to all questions (in this case, we do not know the upper bound on their WTP) or "No" to all questions (in this case, we do not know the lower

bound because WTP can be negative, meaning participants would need to be compensated to accept increased levels of mosquito control).

Data Analysis

Respondent Characteristics

Participant characteristics and corresponding census data for a subset of variables are presented by county in Appendix 1 Table 1. Overall, the average participant was 41 years of age and had lived in their county for ≈22.5 years. A little over half of participants were women. Approximately 37% of respondents identified as Hispanic or Latino/a/x, and 56% identified as a race other than White, with substantial racial and ethnic variation across counties. About a quarter of participants had a high school education or less, while 37% had a bachelor's degree or higher. Regarding income, ≈40% of respondents earned <$40,000 per year, whereas <10% earned >$150,000. A little less than half of participants had children.

To analyze relationships between WTP and respondent characteristics within each county, we use an interval censored regression model in which the dependent variable is the respondent's WTP range and independent variables were gender, race/ethnicity, age, education, income, political ideology, whether or not the respondent knew someone who had had WNV, dengue, or Zika, whether the respondent noticed many mosquitoes outdoors at the time of the survey, and county.

To analyze variation in support for each control method by sociodemographic characteristics, we used ordered logistic regression models for each of the 6 control methods. In these analyses, the dependent variable was the 5-point Likert scale response to the level of support for the method in question and independent variables were gender, race/ethnicity, age, education, income, political ideology, whether or not the respondent knew someone who had had WNV, dengue, or Zika, whether the respondent noticed many mosquitoes outdoors at the time of the survey, and county (Appendix 1 Table 3; Appendix 1 Figure 3).

Discussion

As public health risks from vector-borne disease continue to evolve in the U.S. and around the world, understanding public attitudes and support for control programs can help guide

effective policies and interventions. Results from our survey offer key insights into the public's willingness to pay for expanded mosquito control in Texas, as well as their support for a range of different control measures. Across all three counties surveyed, we found that residents were willing to pay much more for mosquito control than is currently allotted per capita in each of their respective county budgets. On average, participants were willing to pay an additional $53.15 in annual fees for mosquito control while current taxes and grant funding allocate $2 per person per year to vector control in Harris County, $0.27-$0.30 per person in Tarrant County, and $0.05 per person in Hidalgo County. These results suggest that options for scaling up control programs should be explored further. This will require looking at a range of different funding models and approaches and grappling with legal and policy barriers that have constrained control efforts in the past.

In this vein, a 2017 bill introduced in Texas (S.B. 1695) sought to establish mosquito control districts for counties on the Mexico border that either 1) had experienced $\geq$1 locally-transmitted case of Zika, or 2) were located adjacent to a county that had experienced such a case (*18*). These districts would conduct vector surveillance, support county public health work on communicable diseases, and educate the community about vectorborne disease prevention. The bill died in committee, but our results suggest that public opinion is in favor of similar efforts.

In assessing options, Texas officials may find it helpful to examine programs in other states. There is substantial heterogeneity in the scale, scope, and funding of organized vector control activities at the national level (*19*). Legislation varies across states and counties, and policies can either facilitate or prevent revenue collection through property taxes, services charges, or other contracts. California and Florida allow for abatement districts, mostly at the county level, with some county vector control programs having annual budgets of >$10 million per year (*20*). As an example, the Orange County Mosquito and Vector Control District in California has an annual budget of $15,651,880 and a population of 3,190,000, equating to $4.90 per person per year. This is significantly higher than the funding levels in the Texas counties we surveyed, but still lower than our estimated WTP. Property taxes derived from millage rates are not the only revenue source for funding local mosquito and vector control programs. For example, Illinois and Florida both collect fees for tire disposal that fund vector surveillance, control, and research activities (*21–23*).

In terms of control methods, we found that participants were most supportive of lethal traps as a form of mosquito control, which involve neither chemicals nor the release of additional mosquitoes. Ongoing research is shedding light on the effectiveness and feasibility of scaled up mosquito control using such traps (*24*). Participants were also in support of traditional methods of mosquito control (adulticides and larvicides). Consistent with prior work in Key West, Florida (*9*), participants were somewhat more skeptical of methods involving the release of genetically modified mosquitoes or mosquitoes infected with *Wolbachia* bacteria. However, more respondents were in favor of these methods than those in opposition. With intentional community engagement, education, and outreach, these methods may serve as an effective tool in control programs going forward.

Vector control programs have historically been either top-down, bottom-up, or a combination (*25–27*). Top-down approaches led to effective control of *Ae. aegypti* over much of the Americas in the 1950s, but political instability and a lack of sustained efforts caused these programs to fail over time (*1*,*26*,*28*). Currently, many area-wide operational vector control programs are organized at the city or county level. Some counties house multiple mosquito control districts (e.g., Cook Co., Illinois) while others have multi-county programs (e.g., the Metropolitan Mosquito Control District in St. Paul, Minnesota). In some instances, states take on vector control activities: in addition to performing vector-borne disease surveillance, Arizona's state health department runs annual vector control workshops (*29*). In light of the strain that the COVID-19 pandemic is putting on health departments, vector control programs around the country have faced cutbacks, and recently, there has been a call for a national vector surveillance program (*30–32*). A hybrid approach including top-down and bottom-up elements will likely be advantageous. Critically, the funding for this program needs to have bottom-up support through tax-based revenue aligned with the willingness and preferences of local citizens. Support from local communities is key to ensure feasibility and cooperation.

This study is limited in scope to three counties in the large state of Texas, and we cannot generalize our results beyond these counties. While our sampling strategy created a sample that is largely representative of the populations of the selected counties in terms of observable characteristics, the fact that respondents were selected from survey panels recruited for marketing purposes likely results in some unobserved differences between this sample and the general population (e.g., more internet-savvy persons). In addition, stated preference methods

such as the WTP protocol we employed here are subject to hypothetical bias and may result in overestimates of true WTP.

References

1. Newman S. Dallas deploys old weapon in new mosquito fight. 2012 [cited 2019 May 26]. https://www.npr.org/2012/08/19/159013034/dallas-deploys-old-weapon-in-new-mosquito-fight.

2. Luby JP. St. Louis encephalitis. Epidemiol Rev. 1979;1:55–73. PubMed https://doi.org/10.1093/oxfordjournals.epirev.a036214

3. Chung WM, Buseman CM, Joyner SN, Hughes SM, Fomby TB, Luby JP, et al. The 2012 West Nile encephalitis epidemic in Dallas, Texas. JAMA. 2013;310:297–307. PubMed https://doi.org/10.1001/jama.2013.8267

4. Dallas Morning News Staff. Summer of 2012 officially the worst season in Texas for West Nile virus. 2012. Sep 5 [cited 2021 Dec 27]. https://www.dallasnews.com/news/2012/09/06/summer-of-2012-officially-the-worst-season-in-texas-for-west-nile-virus

5. Ropeik D. What's behind the West Nile panic? (Opinion). Dallas Morning News. 2012: Dallas, TX.

6. Limaye VS, Max W, Constible J, Knowlton K. Estimating the health-related costs of 10 climate-sensitive US events during 2012. Geohealth. 2019;3:245–65. PubMed https://doi.org/10.1029/2019GH000202

7. Petersen LR, Fischer M. Unpredictable and difficult to control—the adolescence of West Nile virus. N Engl J Med. 2012;367:1281–4. PubMed https://doi.org/10.1056/NEJMp1210537

8. Ernst KC, Haenchen S, Dickinson K, Doyle MS, Walker K, Monaghan AJ, et al. Awareness and support of release of genetically modified "sterile" mosquitoes, Key West, Florida, USA. Emerg Infect Dis. 2015;21:320–4. PubMed https://doi.org/10.3201/eid2102.141035

9. Adalja, A., Sell, T.K., McGinty, M. and Boddie, C. Genetically modified (GM) mosquito use to reduce mosquito-transmitted disease in the US: a community opinion survey. PLoS Curr 2016 May 25;8:ecurrents.outbreaks.1c39ec05a743d41ee39391ed0f2ed8d3. https://doi.org/10.1371/currents.outbreaks.1c39ec05a743d41ee39391ed0f2ed8d3

10. Joseph A. Florida Keys voters split on genetically modified mosquito trial. Stat 2016 [cited 2020 Nov 6]. https://www.statnews.com/2016/11/08/florida-keys-voters-split-on-genetically-modified-mosquitoes/.

11. British Broadcasting Corporation. Florida mosquitoes: 750 million genetically modified insects to be released. 2020 [cited 2020 Oct 23]. https://www.bbc.com/news/world-us-canada-53856776.

12. Dickinson K, Paskewitz S. Willingness to pay for mosquito control: how important is West Nile virus risk compared to the nuisance of mosquitoes? Vector Borne Zoonotic Dis. 2012;12:886–92. PubMed https://doi.org/10.1089/vbz.2011.0810

13. Dickinson KL, Hayden MH, Haenchen S, Monaghan AJ, Walker KR, Ernst KC. Willingness to pay for mosquito control in Key West, Florida and Tucson, Arizona. Am J Trop Med Hyg. 2016;94:775–9. PubMed https://doi.org/10.4269/ajtmh.15-0666

14. Halasa YA, Shepard DS, Wittenberg E, Fonseca DM, Farajollahi A, Healy S, et al. Willingness-to-pay for an area-wide integrated pest management program to control the Asian tiger mosquito in New Jersey. J Am Mosq Control Assoc. 2012;28:225–36. PubMed https://doi.org/10.2987/12-6243R.1

15. Richards SL, Balanay JAG, Byrd BD, Reiskind MH, Styers DM. Regional survey of mosquito control knowledge and usage in North Carolina. J Am Mosq Control Assoc. 2017;33:331–9. PubMed https://doi.org/10.2987/17-6669.1

16. Langford IH, Bateman IJ, Langford HD. A multilevel modelling approach to triple-bounded dichotomous choice contingent valuation. Environ Resour Econ. 1996;7:197–211.

17. Arrow K, Solow R, Portney PR, Leamer EE, Radner R, Schuman H. Report of the NOAA panel on contingent valuation. Federal Register. 1993;58: 4601–14.

18. State of Texas. Senate Bill 17-1965. An act relating to mosquito control districts established for an urgent public health purpose. 2017 [cited 2021 Dec 27]. https://legiscan.com/TX/text/SB1695/2017

19. Hamer GL. Heterogeneity of mosquito (*Diptera: Culicidae*) control community size, research productivity, and arboviral diseases across the United States. J Med Entomol. 2016;53:485–95. PubMed https://doi.org/10.1093/jme/tjw020

20. Mosquito and Vector Control Association of California (MVCAC). 2020 yearbook. Sacramento, CA: Government of California; 2020.

21. Illinois Environmental Protection Agency. Used tire management fund. 2020 [cited 2020 Nov 24]. https://www2.illinois.gov/epa/topics/waste-management/waste-disposal/used-tires/Pages/fund.aspx.

22. Tabachnick WJ. Florida's state support budget for mosquito control: tough times may undermine Florida public health. 2008 [cited 2020 Nov 24]. https://fmel.ifas.ufl.edu/publication/buzz-words/buzz-words-archive/floridas-state-support-budget-for-mosquito/.

23. Tabachnick WJ. Status of Florida State Aid for Mosquito Control (SAMC). Vero Beach, FL: University of Florida; 2010.

24. Barrera R, Harris A, Hemme RR, Felix G, Nazario N, Muñoz-Jordan JL, et al. Citywide control of *Aedes aegypti* (Diptera: Culicidae) during the 2016 Zika epidemic by integrating community awareness, education, source reduction, larvicides, and mass mosquito trapping. J Med Entomol. 2019;56:1033–46. PubMed https://doi.org/10.1093/jme/tjz009

25. Gubler DJ. *Aedes aegypti* and *Aedes aegypti*–borne disease control in the 1990s: top down or bottom up. Charles Franklin Craig Lecture. Am J Trop Med Hyg. 1989;40:571–8. PubMed https://doi.org/10.4269/ajtmh.1989.40.571

26. Camargo S. History of *Aedes aegypti* eradication in the Americas. Bull World Health Organ. 1967;36:602–3. PubMed

27. Schliessmann DJ. *Aedes aegypti* eradication program of the United States—progress report 1965. Am J Public Health Nations Health. 1967;57:460–5. PubMed https://doi.org/10.2105/AJPH.57.3.460

28. Texas Mosquito Control Association. History of the Texas Mosquito Control Association: the first 25 years (1956–1981). 2020 [cited 2021 January 14]. https://bd043703-f168-425e-90d8-9088d6ede459.filesusr.com/ugd/5282e9_9f5441ddedce4e32b39da908037e2b85.pdf.

29. Arizona Department of Health Services (ADHS). Vector-borne and zoonotic diseases. 2020 [cited 2021 January 13]. https://www.azdhs.gov/preparedness/epidemiology-disease-control/vector-borne-zoonotic-diseases/index.php.

30. US Centers for Disease Control. A national public health framework for the prevention and control of vector-borne diseases in humans. September 28, 2020 [cited 2021 Feb 10]. https://www.cdc.gov/ncezid/dvbd/framework.html.

31. Dykstra L, Gardner A, Spence Beaulieu M. Vector-borne disease: CDC report outlines key steps for prevention and control in U.S. Entomology Today. 2020 Nov 12 [cited 2021 Dec 27]. https://entomologytoday.org/2020/11/12/vector-borne-disease-cdc-report-prevention-control-united-states/

32. Santoro H. COVID-19 is stripping resources from mosquito control programs. Slate. 2020 [cited 2021 Dec 27]. https://slate.com/technology/2020/06/coronavirus-mosquito-vector-control-programs.html

Appendix 1 Table 1. Characteristics of survey participants and comparisons with census characteristics (where available) in study of willingness to pay for mosquito control measures, Texas, United States

Characteristic	Full sample	Harris County		Tarrant County		Hidalgo County	
		Sample	Census	Sample	Census	Sample	Census
n	1,831	610	—	609	—	612	—
Age (median)	37	38	33.9	44	34.8	30	29.6
Gender, %*							
F	56.8	50.0	50.2	50.6	51.0	69.6	52.1
M	42.6	49.3	49.8	48.4	49.0	30.1	47.9
Other/declined	0.7	0.7		1.0		0.3	
Race, %							
Non-Hispanic White	44.6	49.8	28.5	67.2	45.2	16.5	5.6
Hispanic	38.0	20.8	43.7	12.6	29.5	81.0	92.5
Non-Hispanic Black	12.1	20.	18.5	14.8	16.8	1.2	0.4
Non-Hispanic Asian/Pacific Islander	3.2	6.8	7.0	2.2	5.8	0.7	0.9
Non-Hispanic American Indian/Alaska Native/other	2.1	1.8	0.5	3.2	0.6	0.7	0.5
Education, %							
Less than high school	3.3	3.3	17.4	1.8	12.7	5.1	29.9
High school or equivalent	22.2	19.0	25.0	22.8	25.8	25.5	25.3
Some college/2-y degree	37.7	35.4	28.2	35.6	30.5	42.0	28.1
Bachelor's degree or higher	36.7	42.1	29.4	37.7	31.0	27.4	16.7
Annual Income, sample | census%†							
<$40K | <$35K	38.9	33.6	27.5	34.0	22.6	50.5	43.3
$40K–$80K | $35K–$75K	32.1	32.8	30.8	33.3	31.0	30.6	30.5
$80K–$150K | $75–$150K	20.3	22.1	26.1	23.0	30.1	15.5	20.4
>$150,000	7.8	10.7	15.7	9.4	16.3	3.4	5.8
Households with children, %	47.1	46.6	32.8	39.2	31.4	56.1	41.3
Political Ideology							
Liberal	23.5	23.0	—	20.2	—	27.9	—
Moderate	31.6	33.6	—	30.2	—	31.2	—
Conservative	29.0	29.5	—	36.1	—	20.9	—
Know person infected with WNV, Dengue, or Zika, %	13.1	12.1	—	15.1	—	12.3	—
Noticed many mosquitoes outdoors, %	33.2	33.4	—	25.9	—	39.4	—

*Characteristics for which Qualtrics instituted recruitment quotas to align with county census demographics. The gender quota was relaxed for Hidalgo because the response rate had fallen behind the other two counties after a week, resulting in a larger proportion of female participants relative to the proportion of women in the county.
†We categorized our response options differently from the presentation of the income data at data.census.gov.

Appendix 1 Table 2. Interval censored regression results for selected variables in model without covariates (model 1) and with covariates (model 2) in analysis of public willingness to pay for mosquito control, Texas*

Variable	Model 1 result (robust standard error)	Model 2 result (robust standard error)
Constant	55.9 (2.74)*	38.6 (5.93)*
Tarrant County	−3.49 (3.86)	−0.81 (3.86)
Hidalgo County	−3.86 (3.85)	−3.44 (4.55)
Gender: Female		−8.26† (3.28)
Race/Eth: Hispanic		4.24 (4.69)
Race/Eth: Black		2.72 (5.34)
Race/Eth: Other		−12.7† (6.35)
Age: 30–55		0.89 (3.79)
Age: 56+		−5.83 (5.09)
Has Children		5.09 (3.40)
Education: Some college		−0.084 (4.03)
Education: 2 or 4 y degree		5.58 (3.81)
Education: Graduate degree		24.8* (9.31)
Income: $40K–$80K		5.53 (3.79)
Income: $80K–$150K		11.1† (4.58)
Income: More than $150K		24.4* (6.45)
Political ideology: Liberal		11.9* (3.92)
Political ideology: Conservative		1.81 (3.84)
Know someone who had West Nile, Dengue, or Zika		21.3* (4.71)
Notice many mosquitoes outdoors		11.6* (3.38)
Observations	1,831	1,821
AIC	9,226	9,083
Joint significance of county, χ^2 (p value)	1.22 (0.54)	0.58 (0.75)

*p<0.01
†p<0.05

Appendix 1 Table 3. Ordered logistic regression results for level of support of different mosquito control methods, Texas*

	(1)	(2)	(3)	(4)	(5)	(6)
Category	Traps	Adulticide	Larvicide	Sterile Male	Wolbachia	GM
Gender: Female	0.14 (0.091)	−0.068(0.090)	0.016 (0.090)	−0.21‡ (0.090)	−0.22‡ (0.089)	−0.31§ (0.090)
Race/Eth: Hispanic	−0.069 (0.14)	−0.043 (0.14)	−0.078 (0.14)	−0.057 (0.13)	0.019 (0.13)	0.086 (0.13)
Race/Eth: Black	−0.18 (0.15)	0.13 (0.16)	−0.054 (0.14)	−0.43§ (0.15)	−0.090 (0.15)	−0.12 (0.15)
Race/Eth: Other	−0.26 (0.16)	−0.20 (0.16)	−0.30* (0.16)	−0.18 (0.17)	−0.25 (0.18)	−0.17 (0.17)
Age: 30–55	0.25‡ (0.11)	0.19* (0.11)	0.38§ (0.11)	0.14 (0.10)	−0.15 (0.10)	0.080 (0.10)
Age: 56+	−0.19 (0.14)	0.36§ (0.13)	0.61§ (0.14)	0.48§ (0.13)	0.11 (0.13)	0.54§ (0.13)
Has Children	−0.057 (0.096)	−0.016 (0.097)	0.15 (0.098)	0.041 (0.095)	−0.073 (0.093)	0.087 (0.093)
Education: Some college	0.16 (0.11)	0.25‡ (0.11)	0.24‡ (0.11)	−0.013 (0.11)	−0.12 (0.11)	−0.12 (0.11)
Education: 2 or 4 y degree	0.13 (0.11)	0.092 (0.11)	0.29† (0.11)	0.24‡ (0.11)	0.080 (0.11)	0.10 (0.11)
Education: Graduate degree	0.26 (0.28)	0.36 (0.26)	0.52* (0.27)	0.38 (0.25)	0.040 (0.23)	0.19 (0.23)
Income: $40K– $80K	0.081 (0.10)	0.052 (0.10)	0.014 (0.11)	0.012 (0.10)	−0.011 (0.10)	−0.054 (0.10)
Income: $80K – $150K	0.38† (0.13)	0.0066 (0.13)	0.039 (0.13)	−0.053 (0.13)	−0.10 (0.13)	−0.11 (0.13)
Income: More than $150K	0.45‡ (0.19)	0.50† (0.18)	0.33* (0.18)	0.28 (0.20)	0.20 (0.19)	0.11 (0.19)
Political ideology: Liberal	0.047 (0.11)	0.054 (0.11)	0.068 (0.11)	0.13 (0.11)	0.12 (0.10)	0.18* (0.11)
Political ideology: Conservative	0.15 (0.11)	0.29† (0.11)	0.18 (0.11)	0.025 (0.11)	0.20* (0.11)	0.074 (0.11)
Know someone who had West Nile, Dengue, or Zika	0.16 (0.14)	0.45† (0.15)	0.21 (0.14)	0.21 (0.14)	0.24* (0.14)	0.38† (0.14)
Notice many mosquitoes outdoors	0.15 (0.10)	0.48† (0.10)	0.32† (0.099)	0.12 (0.096)	0.17* (0.095)	0.092 (0.096)
Tarrant County	0.22‡ (0.11)	−0.24‡ (0.11)	0.094 (0.10)	0.0022 (0.11)	−0.083 (0.11)	−0.087 (0.11)
Hidalgo County	0.18 (0.13)	−0.019 (0.14)	−0.022 (0.14)	0.093 (0.12)	0.054 (0.12)	0.0095 (0.13)
Observations	1,821	1,821	1,821	1,821	1,821	1,821
Pseudo R^2¶	0.01	0.02	0.02	0.01	0.01	0.01
Joint significance of county: χ^2 (p value)	4.34 (0.11)	5.41 (0.07)	1.13 (0.57)	0.65 (0.72)	1.30 (0.52)	0.87 (0.65)

*Robust standard errors in parentheses. The dependent variable in each of these ordered logistic regressions is the level of support for each specified control method (1=Strongly Oppose, 2= Oppose, 3=Neutral/No Opinion, 4=Support, 5=Strongly Support), and rows show regression coefficients and standard errors for each independent variable. . GM, genetic modification.
†p<0.01
‡p<0.05
§p<0.1
¶Goodness-of-fit measure for nonlinear regression (logit) model.

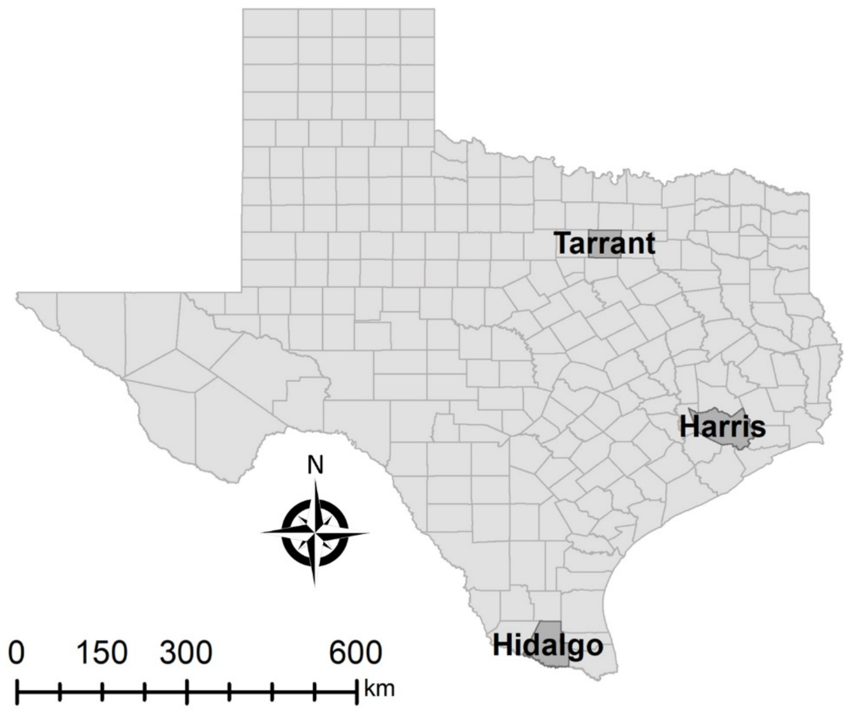

Appendix 1 Figure 1. Map of Texas showing Harris, Tarrant, and Hidalgo counties, in which residents were surveyed for willingness to pay for mosquito-control measures.

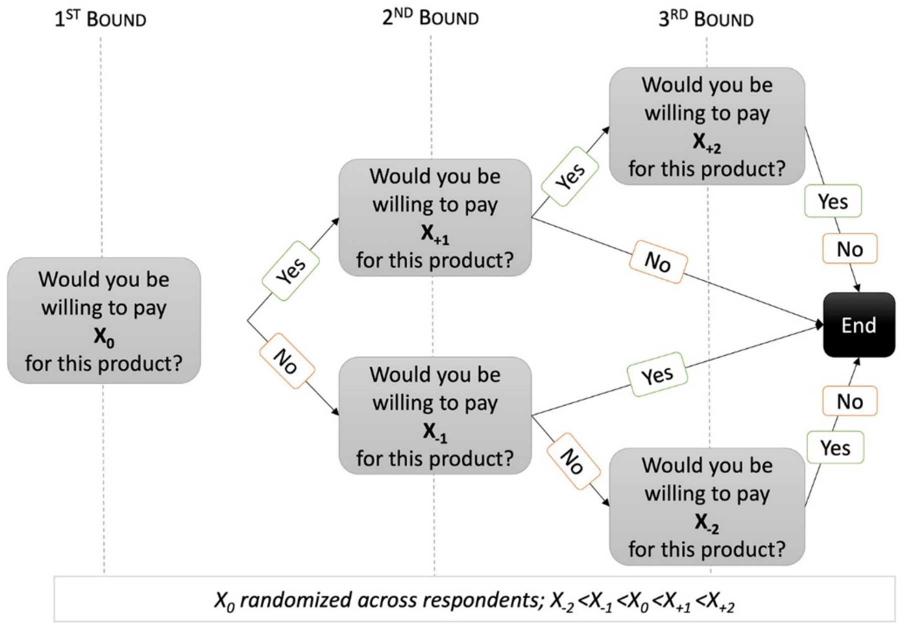

Appendix 1 Figure 2. Triple-bounded dichotomous-choice framework to measure willingness-to-pay for mosquito control, Texas, US.

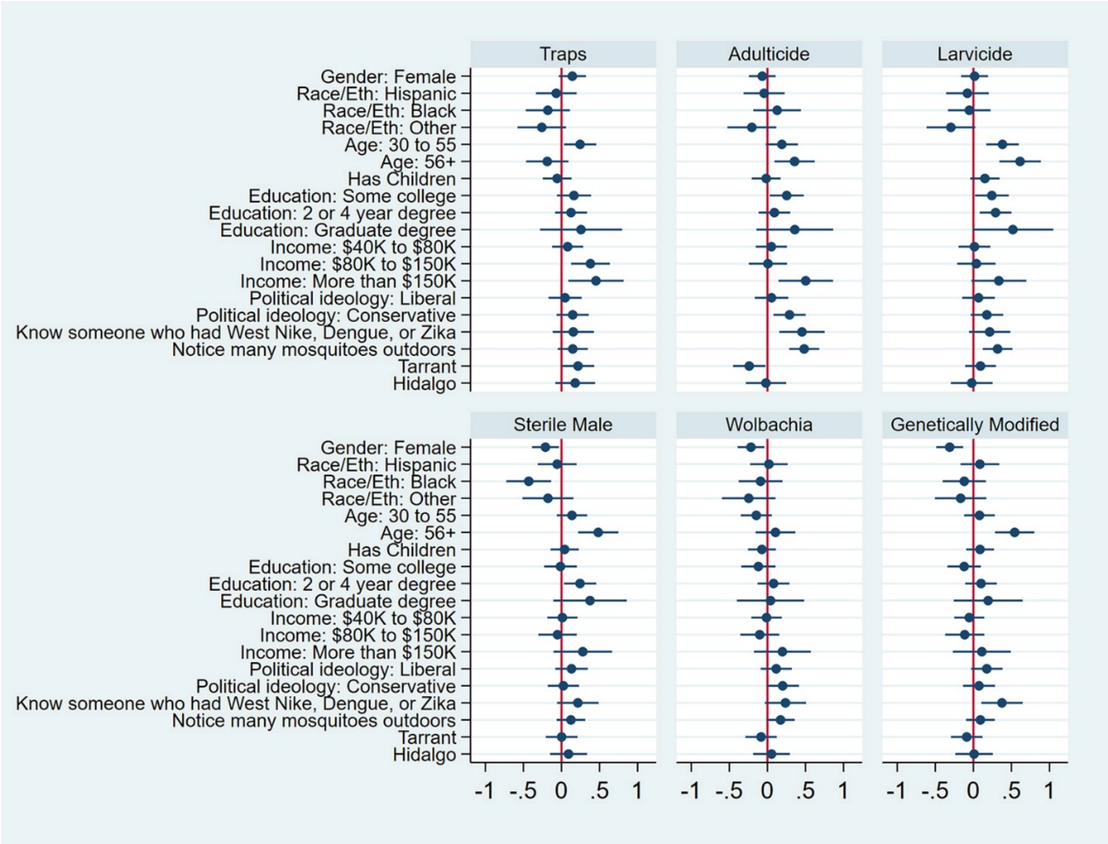

Appendix 1 Figure 3. Ordered logistic regression results showing variation in support for mosquito control methods by individual characteristics. Dots indicate point estimates and lines indicate 95% CI. Red line represents the reference category, e.g., male sex, non-Hispanic white race/ethnicity, respondents under age 30, respondents without children (Appendix 1 Table 1).
