# Supplementary material for: Public Acceptance of and Willingness to Pay for Mosquito Control, Texas, USA
Source: Emerg Infect Dis. 2022 Feb;28(2):425–8. doi: 10.3201/eid2802.210501 (PMC8798704; doi:10.3201/eid2802.210501)
Supplement: Appendix 2 — Survey instrument and fact sheets used in study of willingness to pay for and acceptance of mosquito control, Texas, USA. [file 21-0501-Techapp-s2.pdf]

Article DOI: https://doi.org/10.3201/eid2801.210501

Public Acceptance of and Willingness to Pay for Mosquito Control, Texas

Appendix 2

The following pages show a survey given to residents of Harris, Tarrant, and Hidalgo Counties in Texas, USA, to determine public attitudes toward and willingness to pay for mosquito control, regions with varying risk for mosquitoborne pathogens, socioeconomic conditions, and current mosquito control practices.

English

Informed Consent

You have been invited to take a research survey. This form provides you with information about the survey and how this information will help our research study. Please read the information below. If you have questions about anything you don't understand before deciding whether or not to participate, please contact the study investigator, Dr. Katherine Dickinson of the Colorado School of Public Health at (303) 724-4093 or email her at katherine.dickinson@cuanschutz.edu.

Why is this survey being done?
To learn more about mosquitoes and mosquito control in this area. It is important that the opinions and attitudes of Texas residents dealing with mosquitoes and their diseases be understood and considered to develop more effective surveillance and control programs. Therefore, final survey results will be made available to local, state, and federal groups considering mosquito control programs.

What happens if I take this survey?
Your experiences and opinions surrounding mosquito-borne disease and control will contribute to a valuable data set that will help determine the burden of mosquitoes in this area and how they should be managed. This survey will take approximately 15 minutes.

What are the possible discomforts or risks?
There are no foreseeable risks associated with your participation in this survey.

What are the possible benefits of the study?
There are no known benefits directly resulting from your participation in this survey.

Who is paying for this study?
The US Centers for Disease Control and Prevention.

Will I be paid for being in the study? Will I have to pay for anything?

No, you will not be paid, and it will not cost you anything to participate in this survey.

Is my participation voluntary?
Yes. You have the right to choose not to participate in this study. If you choose to participate, you have the right to stop at any time, or to skip any questions you do not wish to answer. If you refuse or decide to withdraw later, you will not lose any benefits or rights to which you are entitled.

Who do I call if I have questions?
The researchers leading this study are Dr. Katherine Dickinson (Colorado School of Public Health) and Dr. Gabriel Hamer (Texas A&M University). If you have questions, you may call Dr. Dickinson at (303) 724-4093 or email her at katherine.dickinson@cuanschutz.edu.

You may have questions about your rights as a participant in this study. You can also call the Colorado Multiple Institutional Review Board (COMIRB) at 303-724-1055.

Who will see my research information?
We will make every effort to maintain the confidentiality of the survey data. No information about individuals who participate in the study will ever be published; all results will be presented in summary form. All records and data will be kept secure to the best of our ability. Individual data may be viewed by the following people:

- *Federal agencies that monitor human subject research*
- *Human Subject Research Committee*
- *The group doing the study*
- *The group paying for the study*
- *Regulatory officials from the institution where the research is being conducted who want to make sure the research is safe*

Survey results will be shared in meetings, reports, and published articles. Your name will be kept private when information is presented.

Agreement to take this survey

I have read the previous description about the survey. I understand the possible risks and benefits of this survey. I know that taking this survey is voluntary. I choose to take this survey.

Yes

No

Sociodemographic Information

We are almost done with this survey. We would just like to ask a few questions about you that will help us better understand the results!

What is your age?

What is your gender?

Male

Female

Non-binary/third gender

Prefer to self-describe:

Prefer not to say

Do you consider yourself Hispanic or Latino?

Yes

No

Prefer not to answer

How would you classify yourself in regards to race?

American Indian/Alaska Native

Asian/Pacific Islander

Black/African American

White

Multi-racial

Other

Prefer not to answer

When it comes to politics, do you usually think of yourself as:

Very Liberal

Liberal

Moderate

Conservative

Very Conservative

Have not considered this/prefer not to answer

What is your political affiliation?

Democrat

Republican

Libertarian

Green Party

Unaffiliated/Independent

Other

Prefer not to answer

What is your marital status?

Married

Cohabitating/common law

Single

Divorced/Separated

Widowed

Prefer not to answer

What is the highest level of education you have completed?

Less than high school

High school graduate

Some college

2 year degree

4 year degree

Master's degree

Professional degree

Doctorate

Prefer not to answer

What was your household's income last year?

Less than $20,000

$20,000-$39,999

$40,000-$59,999

$60,000-$79,999

$80,000-$99,999

$100,000-$149,999

$150,000-$199,999

More than $200,000

Prefer not to answer

Respondent Type Screening

Please enter your county.

Harris County

Tarrant County

Hidalgo County

Please enter your zip code.

Household Characteristics

How many adults (over the age of 18) live in your home?

How many children live in your home?

How many years have you lived in Harris County?

How many years have you lived in Hidalgo County?

How many years have you lived in Tarrant County?

Do you have air conditioning?

Yes

No

How often do you use your air conditioning during the summer months?

Every day

Multiple times per week

About once a week

Less than once a week

Other

Do your windows or outside doors have screens?

Yes, all of them

Yes, some of them

No, none of them

Perceived impacts of mosquitoes on quality of life

On a typical weekend or non-working day at this time of year, how many hours do you spend outdoors?

0-2

2-4

4-6

6-8

8+

On a typical weekend or non-working day at this time of year, how many hours do your kids spend outdoors (if applicable)?

0-2

2-4

4-6

6-8

8+

What do you think about mosquitoes in your community?

1 - Not a problem

2 - Small problem

3 - Moderate problem

4 - Big problem

5 - Very big problem

Currently, do you notice mosquitoes when you are outdoors in your neighborhood?

None

Very few

Moderate amount

Quite a few

Very many

Not applicable

Have you noticed mosquitoes biting you in the past week OUTSIDE your home?

Yes

No

Have you noticed mosquitoes biting you in the past week INSIDE your home?

Yes

No

In the past month, have mosquitoes caused you to avoid or shorten the amount of time you spent doing any of the following activities? Select all that apply.

Cooking out

Dining outside or picnicking

Gardening

Yard or home maintanence

Walking in the neighborhood

Playing in the yard with a child

Talking with neighbors

[ ] Other

None

During which months do mosquitoes bother you the most?

January

February

March

April

May

June

July

August

September

October

November

December

Please list any diseases that are spread by mosquitoes in this area.

Have you heard of West Nile Virus?

Yes

No

How concerned are you about West Nile Virus in your area?

1 - Not at all concerned

2 - Slightly concerned

3 - Somewhat concerned

4 - Very concerned

5 - Extremely concerned

Don't know / Not sure

Have you heard of Dengue virus?

Yes

No

How concerned are you about Dengue virus in your area?

1 - Not at all concerned

2 - Slightly concerned

3 - Somewhat concerned

4 - Very concerned

5 - Extremely concerned

Don't know / Not sure

Have you heard of Zika?

Yes

No

How concerned are you about Zika in your area?

1 - Not at all concerned

2 - Slightly concerned

3 - Somewhat concerned

4 - Very concerned

5 - Extremely concerned

Don't know / Not sure

Have you known anyone personally who has had any of the following mosquito-borne diseases?

	Yes	No	Don't know / Not sure
West Nile Virus	O	O	O
Dengue Virus	O	O	O
Zika	O	O	O

Mosquito Avoidance

Which of the following methods do you use to avoid being bitten by mosquitoes? Select all that apply.

Using repellent

Burning citronella candles

Wearing long sleeves

Draining standing water

Removing containers (tires, bottles, plant saucers, bird baths) that collect water

Calling mosquito control

Burning coils

Burning "tiki torches"

Spraying insecticide

Staying indoors

Plant mosquito-repellent plants (ex. lemon balm, rosemary)

Fans

None

Other

About how much do you think you spent on products to avoid mosquitoes in the past month?

$0

$1-$5

$5-$10

$10-$15

$15-$20

$20-$30

More than $30

Rating Current Programs

How would you rate the current mosquito control programs in your area?

1 - Not at all effective

2 - Slightly effective

3 - Somewhat effective

4 - Very effective

5 - Extremely effective

6 - I am not aware of any control programs in my area

In your opinion, should mosquito control efforts in your area:

Be expanded

Be reduced

Be maintained at current levels

Not sure

Do you have any concerns about the possible side effects of mosquito control in your county? Select all that apply.

I have concerns about the effects of insecticides on the environment, including plants, animals, and other insects

I have concerns about the effects of insecticides on human health

I have concerns about mosquitoes developing resistance to insecticides used in mosquito control (i.e. control methods become ineffective)

I have concerns about the possible impacts of using genetically modified mosquitoes for mosquito control

I have concerns about mosquito control invading my privacy (too much government)

I don't have any concerns about mosquito control

Other

In general, please indicate your level of support for a county-wide mosquito control program:

I am very supportive of a county-wide mosquito control program

I am somewhat supportive of a county-wide mosquito control program

I don't really care if there is a mosquito control program or not

I am somewhat opposed to a county-wide mosquito control program

I am strongly opposed to a county-wide mosquito control program

Don't know/not sure

Willingness to Pay

The Harris County Public Health Mosquito & Vector Control Division currently coordinates all mosquito control activities in this county including the city of Houston. Some municipalities also collaborate with Harris County to enhance control. Activities include:

- Surveillance (trapping mosquitoes to see how many and what types of mosquitoes are present in different areas) and testing for mosquito-borne diseases;

- Mosquito control using adulticides (spraying chemicals to kill adult mosquitoes) and minimal larvicides (treating mosquito breeding sites to kill immature mosquitoes);

- Insecticide resistance testing, mosquito inspections, virology testing, bird surveillance, alternative mosquito control applied research, other vector surveillance (i.e. ticks and kissing bugs);

- Education and outreach.

These activities are focused on mosquitoes that can spread West Nile virus, St. Louis encephalitis, and more recently, mosquitoes that can transmit dengue, Zika, and chikungunya viruses. Funding for mosquito control comes mainly from taxes and bonds, and federal grants during emergencies. The total annual budget for mosquito control in Harris County is about $6 to 8 million, or roughly $2 per person per year.

The Tarrant County Public Health Department currently coordinates mosquito control activities in this county, and some municipalities also carry out their out mosquito control activities. These activities include:

- Surveillance (trapping mosquitoes to see how many and what types are present in different areas); testing people and mosquitoes for disease;

- Mosquito control using adulticides (spraying chemicals to kill primarily disease-carrying adult mosquitoes) and larvicides (treating mosquito breeding sites to kill immature mosquitoes);

- Education and outreach.

Mosquito control activities are largely focused on trapping and testing Culex mosquitoes that can spread West Nile Virus and St. Louis encephalitis, and spraying the area if positive. More recently, the county has also been doing surveillance for mosquitoes that can transmit dengue, Zika, and chikungunya viruses and testing people for these diseases.

Funding for mosquito control comes from the county's general tax fund, as well as grants. The annual budget for mosquito control in Tarrant County is about $700,000 or roughly $0.27-$0.30 per person, per year. This estimate does not include the funds that individual cities in Tarrant County have in their budgets for mosquito control activities.

Mosquito control activities in Hidalgo County are mainly run by each city's health department. Hidalgo County Health and Human Services supports the cities and helps with mosquito control in cities without a health department. Depending on the city, activities that are conducted may include:

- Surveillance (trapping mosquitoes to see how many and what types are present in different areas)

- Mosquito control using adulticides (spraying chemicals to kill adult mosquitoes) and larvicides (treating mosquito breeding sites to kill immature mosquitoes)

- Education and outreach.

Mosquito control activities in the past have focused on mosquitoes that can transmit West Nile Virus and dengue. More recently, the county has also begun conducting targeted surveillance for mosquitoes that can transmit Zika and chikungunya viruses.

The county-wide vector control program has an annual budget of about $10,000 and covers the 200,000 people in Hidalgo County that do not fall under one of the other 23 city vector departments in the area. This equates to about $0.05 per person each year.

We're now going to ask you about whether you'd be willing to pay to increase mosquito control at the county level. Suppose that there were a proposal on the next election ballot to expand mosquito control across the county. If the proposal passes, the number of mosquitoes in this area would be cut in half. To fund this expansion, your household and others in the county

would be charged an annual fee. The next questions will ask you whether or not you would vote in favor of this ballot measure.

Please consider these questions and answer honestly. Your responses may inform future policy in this area. Any response (yes, no, or not sure) is valid. Some reasons people might support this proposal are that they value mosquito control and think it is worth the money to reduce mosquitoes. Some reasons people might oppose this proposal are that they think the fee is too high or do not think it's worth the money to expand mosquito control activities.

WTP Block 1

Suppose that there were a proposal on the next election ballot to expand mosquito control across the county. If the proposal passes, the number of mosquitoes in this area would be cut in half. To fund this expansion, your household and others in the county at your income level would be charged a fee of $25 once per year. Would you support this proposal?

No

Yes

Not sure

Now suppose that instead of $25, the fee for this program (which would still reduce the number of mosquitoes by half) was $50 per year. Would you still support this proposal?

No

Yes

Not sure

Now suppose the fee for this same program were even higher: $100 per year. Would you still support this proposal?

No

Yes

Not sure

Now suppose that instead of $25, the fee for this program (which would still reduce the number of mosquitoes by half) was $10 per year. Would you support this proposal?

No

Yes

Not sure

Now suppose the fee for this same program were even lower: $5 per year. Would you support this proposal?

No

Yes

Not sure

We are interested in knowing why you indicated that you are not willing to pay for mosquito control programs. Please select all reasons that apply.

Mosquitoes don't bother me

I don't trust government intervention

I can't afford it/it's too expensive

I don't like paying additional fees

I don't think the program would be successful

Other

Don't know

We are interested in why you indicated that you are willing to pay for mosquito control programs. Please choose all reasons that apply.

I think mosquitoes are a nuisance

I'm worried about disease risk

I think the program would be successful

I want to show support for such a program

Other

Don't know

We are interested in why you are unsure whether or not you would be willing to pay for mosquito control programs. Please choose all reasons that apply.

I would need more information

I did not understand the questions

[ ] Other

Don't know

What additional information would you need in order to make a decision?

[ ]

To what extent do you believe that these survey results will be taken into consideration by county policymakers making decisions about mosquito control programs?

I don't think policymakers will consider these surveys

I think it is unlikely that policymakers will consider these surveys

I don't know whether or not policymakers will consider these surveys

I think it is likely that policymakers will consider these surveys

I think policymakers will consider these surveys

WTP 5 ($100)

Suppose that there were a proposal on the next election ballot to expand mosquito control across the county. If the proposal passes, the number of mosquitoes in this area would be cut in half. To fund this expansion, your household and others in the county at your income level would be charged a fee of $100 once per year. Would you support this proposal?

No

Yes

Not sure

Now suppose that instead of $100, the fee for this program (which would still reduce the number of mosquitoes by half) was $200 per year. Would you still support this proposal?

No

Yes

Not sure

Now suppose the fee for this same program were even higher: $400 per year. Would you still support this proposal?

No

Yes

Not sure

Now suppose that instead of $100, the fee for this program (which would still reduce the number of mosquitoes by half) was $50 per year. Would you support this proposal?

No

Yes

Not sure

Now suppose the fee for this same program were even lower: $25 per year. Would you support this proposal?

No

Yes

Not sure

We are interested in knowing why you indicated that you are not willing to pay for mosquito control programs. Please select all reasons that apply.

Mosquitoes don't bother me

I don't trust government intervention

I can't afford it/it's too expensive

I don't like paying additional fees

I don't think the program would be successful

Other

Don't know

We are interested in why you indicated that you are willing to pay for mosquito control programs. Please choose all reasons that apply.

I think mosquitoes are a nuisance

I'm worried about disease risk

I think the program would be successful

I want to show support for such a program

Other

Don't know

We are interested in why you are unsure whether or not you would be willing to pay for mosquito control programs. Please choose all reasons that apply.

I would need more information

I did not understand the questions

Other

Don't know

What additional information would you need in order to make a decision?

To what extent do you believe that your vote and that of other survey participants will be taken into consideration by county policymakers?

I don't think policymakers will consider these votes

I think it is unlikely that policymakers will consider these votes

I don't know whether or not policymakers will consider these votes

I think it is likely that policymakers will consider these votes

I think policymakers will consider these votes

WTP 4 ($50)

Suppose that there were a proposal on the next election ballot to expand mosquito control across the county. If the proposal passes, the number of mosquitoes in this area would be cut in half. To fund this expansion, your household and others in the county at your income level would be charged a fee of $50 once per year. Would you support this proposal?

No

Yes

Not sure

Now suppose that instead of $50, the fee for this program (which would still reduce the number of mosquitoes by half) was $100 per year. Would you still support this proposal?

No

Yes

Not sure

Now suppose the fee for this same program were even higher: $200 per year. Would you still support this proposal?

No

Yes

Not sure

Now suppose that instead of $50, the fee for this program (which would still reduce the number of mosquitoes by half) was $25 per year. Would you support this proposal?

No

Yes

Not sure

Now suppose the fee for this same program were even lower: $10 per year. Would you support this proposal?

No

Yes

Not sure

We are interested in knowing why you indicated that you are not willing to pay for mosquito control programs. Please select all reasons that apply.

Mosquitoes don't bother me

I don't trust government intervention

I can't afford it/it's too expensive

I don't like paying additional fees

I don't think the program would be successful

Other

Don't know

We are interested in why you indicated that you are willing to pay for mosquito control programs. Please choose all reasons that apply.

I think mosquitoes are a nuisance

I'm worried about disease risk

I think the program would be successful

I want to show support for such a program

Other

Don't know

We are interested in why you are unsure whether or not you would be willing to pay for mosquito control programs. Please choose all reasons that apply.

I would need more information

I did not understand the questions

Other

Don't know

What additional information would you need in order to make a decision?

To what extent do you believe that your vote and that of other survey participants will be taken into consideration by county policymakers?

I don't think policymakers will consider these votes

I think it is unlikely that policymakers will consider these votes

I don't know whether or not policymakers will consider these votes

I think it is likely that policymakers will consider these votes

I think policymakers will consider these votes

WTP Block 3 ($10)

Suppose that there were a proposal on the next election ballot to expand mosquito control across the county. If the proposal passes, the number of mosquitoes in this area would be cut in half. To fund this expansion, your household and others in the county at your income level would be charged a fee of $10 once per year. Would you support this proposal?

No

Yes

Not sure

Now suppose that instead of $10, the fee for this program (which would still reduce the number of mosquitoes by half) was $20 per year. Would you still support this proposal?

No

Yes

Not sure

Now suppose the fee for this same program were even higher: $40 per year. Would you still support this proposal?

No

Yes

Not sure

Now suppose that instead of $10, the fee for this program (which would still reduce the number of mosquitoes by half) was $5 per year. Would you support this proposal?

No

Yes

Not sure

Now suppose the fee for this same program were even lower: $2 per year. Would you support this proposal?

No

Yes

Not sure

We are interested in knowing why you indicated that you are not willing to pay for mosquito control programs. Please select all reasons that apply.

Mosquitoes don't bother me

I don't trust government intervention

I can't afford it/it's too expensive

I don't like paying additional fees

I don't think the program would be successful

Other

Don't know

We are interested in why you indicated that you are willing to pay for mosquito control programs. Please choose all reasons that apply.

I think mosquitoes are a nuisance

I'm worried about disease risk

I think the program would be successful

I want to show support for such a program

Other

Don't know

We are interested in why you are unsure whether or not you would be willing to pay for mosquito control programs. Please choose all reasons that apply.

I would need more information

I did not understand the questions

Other

Don't know

What additional information would you need in order to make a decision?

To what extent do you believe that your vote and that of other survey participants will be taken into consideration by county policymakers?

I don't think policymakers will consider these votes

I think it is unlikely that policymakers will consider these votes

I don't know whether or not policymakers will consider these votes

I think it is likely that policymakers will consider these votes

I think policymakers will consider these votes

WTP Block 2 ($5)

Suppose that there were a proposal on the next election ballot to expand mosquito control across the county. If the proposal passes, the number of mosquitoes in this area would be cut in half. To fund this expansion, your household and others in the county at your income level would be charged a fee of $5 once per year. Would you support this proposal?

No

Yes

Not sure

Now suppose that instead of $5, the fee for this program (which would still reduce the number of mosquitoes by half) was $10 per year. Would you still support this proposal?

No

Yes

Not sure

Now suppose the fee for this same program were even higher: $20 per year. Would you still support this proposal?

No

Yes

Not sure

Now suppose that instead of $5, the fee for this program (which would still reduce the number of mosquitoes by half) was $2 per year. Would you support this proposal?

No

Yes

Not sure

Now suppose the fee for this same program were even lower: $1 per year. Would you support this proposal?

No

Yes

Not sure

We are interested in knowing why you indicated that you are not willing to pay for mosquito control programs. Please select all reasons that apply.

Mosquitoes don't bother me

I don't trust government intervention

I can't afford it/it's too expensive

I don't like paying additional fees

I don't think the program would be successful

Other

Don't know

We are interested in why you are unsure whether or not you would be willing to pay for mosquito control programs. Please choose all reasons that apply.

I would need more information

I did not understand the questions

Other

Don't know

We are interested in why you indicated that you are willing to pay for mosquito control programs. Please choose all reasons that apply.

I think mosquitoes are a nuisance

I'm worried about disease risk

I think the program would be successful

I want to show support for such a program

Other

Don't know

What additional information would you need in order to make a decision?

To what extent do you believe that your vote and that of other survey participants will be taken into consideration by county policymakers?

I don't think policymakers will consider these votes

I think it is unlikely that policymakers will consider these votes

I don't know whether or not policymakers will consider these votes

I think it is likely that policymakers will consider these votes

I think policymakers will consider these votes

Description of Mosquito Control Options

There are many different types of mosquitoes in Texas. Some types of mosquitoes can transmit diseases. Culex is one of the main types of mosquitoes in this area that can spread diseases to humans, especially West Nile virus. More recently, health departments have also been monitoring some Aedes mosquitoes which can spread dengue, Zika and chikungunya viruses.

Some control methods specifically target disease-carrying mosquitoes. Some are more effective against Culex mosquitoes, and others are more effective against Aedes mosquitoes. All mosquitoes that feed on humans can be a nuisance!

Please read the information below about different types of control methods. We will then ask you some questions about what you think of these methods.

Adulticides

This method involves spraying insecticides to reduce the mosquito population by killing adult mosquitoes. Adulticides can be applied using handheld sprayers, or sprayers mounted on a backpack, a truck or an airplane.

Have adulticides been tested?

Yes.

Have adulticides been used in Texas?

Yes, they are widely used across the state.

Which types of mosquitoes are targeted by this method?

Nuisance mosquitoes and mosquitoes that carry disease are targeted.

Often, mosquito control entities will use adulticides when they find a mosquito that is carrying West Nile or another virus.

Recent testing in the U.S. has shown that some adulticides can successfully kill mosquitoes that can carry Zika, chikungunya and dengue.

Are adulticides expensive?

Adulticides are widely used because they are low-cost and continue to be highly effective over time.

Are adulticides harmful to human health and/or the environment?

Adulticides can be dangerous at high levels of exposure, like in the event of a spill.

It is possible for people to breathe in small amounts of adulticides when a spraying takes place. People should stay inside and close doors and windows during spraying.

Some adulticides are toxic to bees and fish, so they are often applied only at certain times and places in order to protect wildlife.

Larvicides

Larvicides kill immature mosquitoes in their water habitat before they reach the flying adult stage.

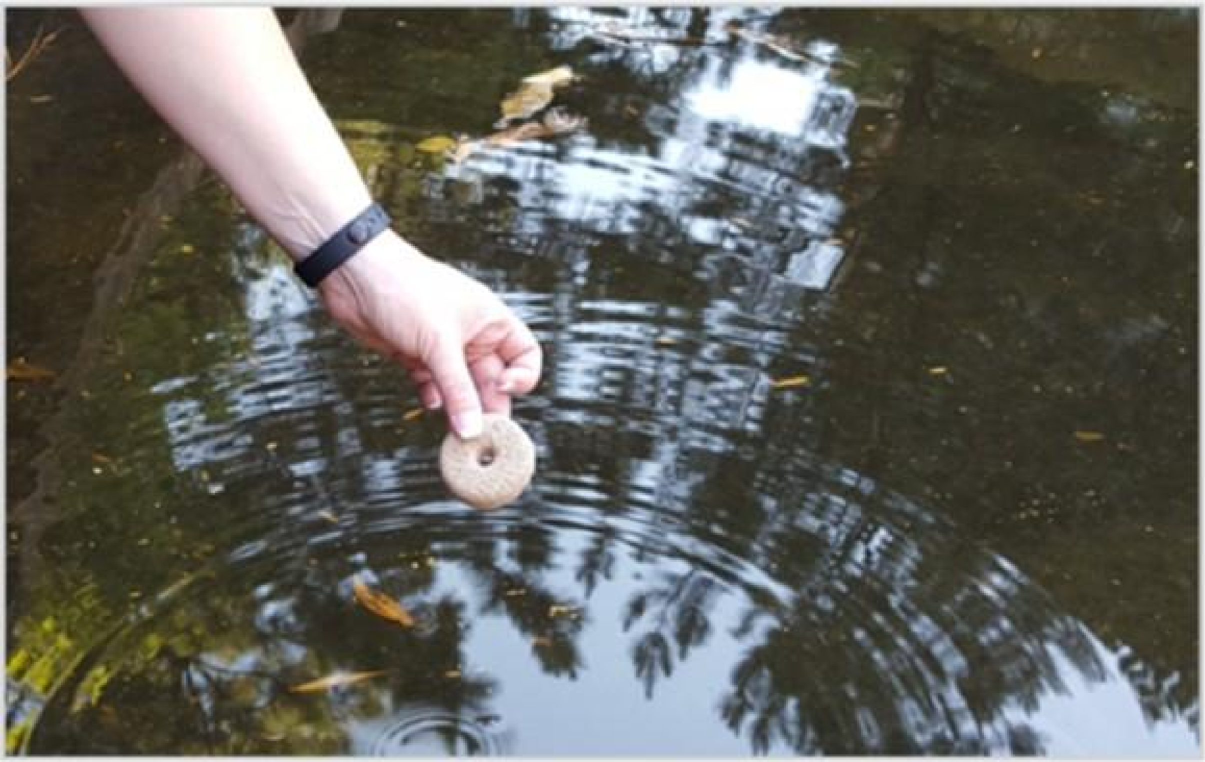

Have larvicides been tested?

Yes.

Have larvicides been used in Texas?

Yes.

Which mosquitoes does this method target?

Larvicides can target mosquitoes such as Culex and Aedes, which breed in containers. They can also be applied over wider areas to target nuisance mosquitoes that breed in flood water and salt marshes.

Are larvicides expensive?

Larvicides are generally the lowest-cost and most effective way to control mosquitoes since they kill mosquitoes before they can fly and bite people.

Are larvicides harmful to human health and/or the environment?

Larvicides have not been shown to be harmful to humans, but anyone using these products should wear appropriate protection to avoid skin and eye irritation.

Some larvicides can be toxic to fish and other aquatic life.

Mosquito Kill Traps

Different kinds of traps attract different types of adult mosquitoes. Once they enter the trap, mosquitoes can't escape and they die.

One type of trap attracts female mosquitoes into a water-filled bucket, where the mosquitoes want to lay eggs, but they get stuck to a glue before they reach the water.

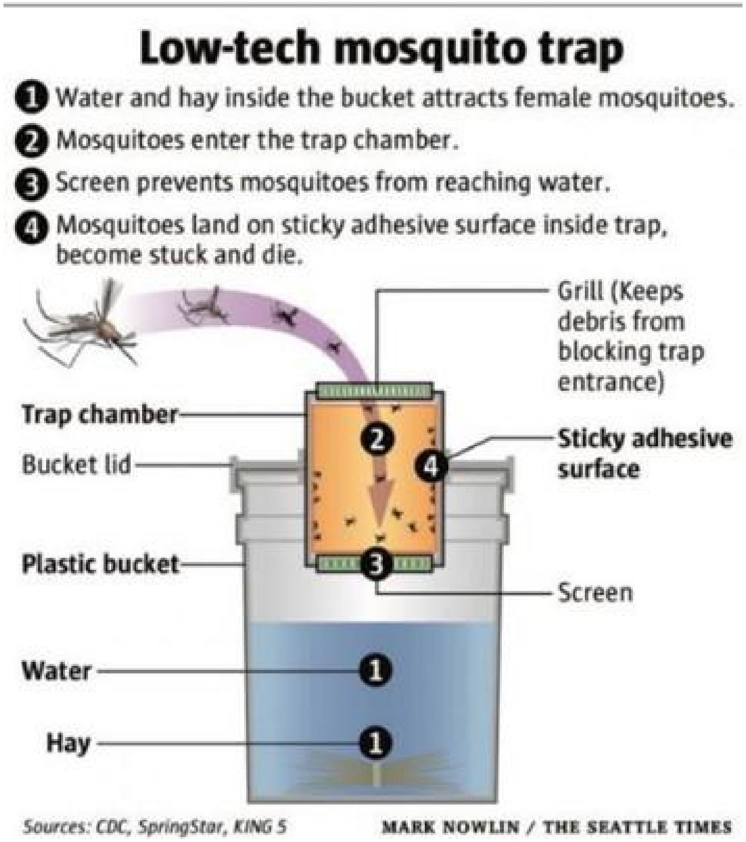

Have these traps been tested?

Yes, they have been tested for safety and effectiveness and are still being researched. Although the traps kill only a few mosquitoes per week, it has been shown that placing 3 traps in most of the homes in a community can greatly reduce the mosquito population.

Have traps been used in Texas?

Yes.

Which mosquitoes does this method target?

These traps mainly target the container-breeding mosquitoes (*Aedes*) that can carry West Nile virus, dengue, Zika, and chikungunya.

Are traps expensive?

The price ranges from $15 to $40 per unit. Traps require maintenance every month or two.

Are traps harmful to human health and/or the environment?

No.

Mass Release of Modified Mosquitoes

Some techniques involve releasing many mosquitoes that have been modified in a lab to reduce the population of mosquitoes and/or prevent the spread of disease. Generally, these mosquitoes are non-biting males.

One method involves releasing sterile male mosquitoes. When they mate with wild mosquitoes, they cannot reproduce, which shrinks the mosquito population over time.

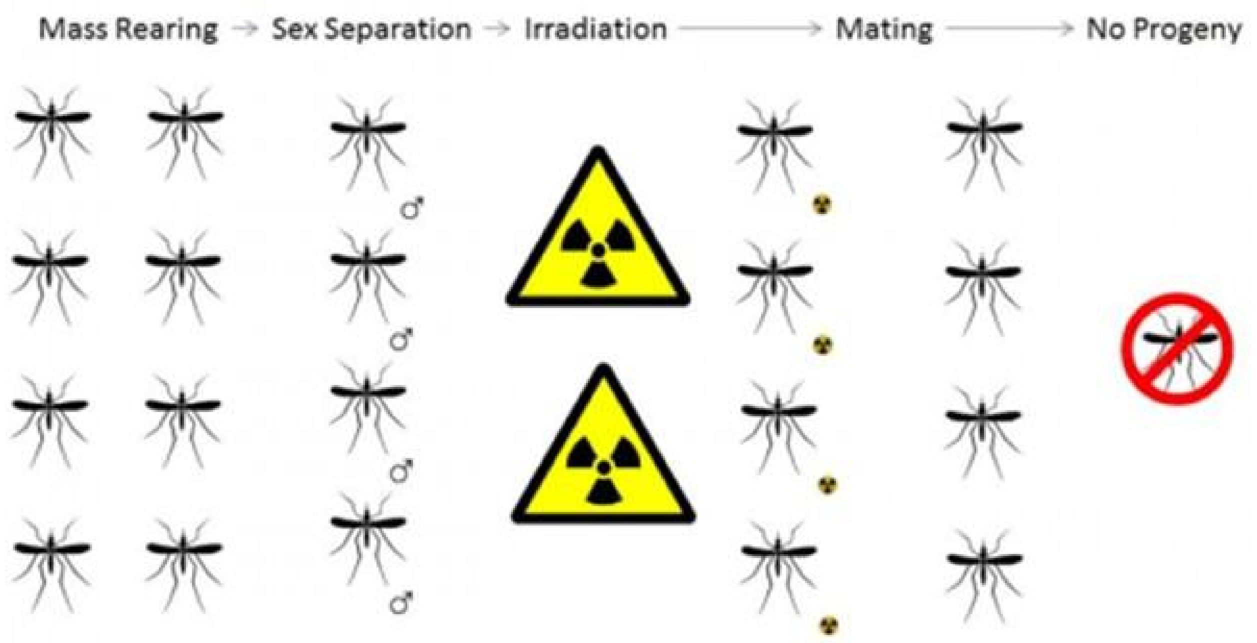

Have sterile male mosquitoes been tested?

Yes, and research is still being done on this method.

Have sterile male mosquitoes been released in Texas?

No, but releases are planned along the Texas-Mexico border soon.

Which mosquitoes does this method target?

This method has been tested targeting *Aedes* mosquitoes that can carry West Nile virus, dengue, Zika, and chikungunya.

Is it expensive to release sterile male mosquitoes?

This approach is still being tested and the cost of an area-wide control program using this method has not been established yet.

Are sterile male mosquitoes harmful to human health and/or the environment?

So far, the results of testing suggest they are not harmful.

Mass Release of Genetically Modified (GM) Mosquitoes

Genetically modified mosquitoes can also be mass released. Once the genetically modified mosquitoes mate with wild ones, very few of the offspring survive. The company Oxitec uses this method.

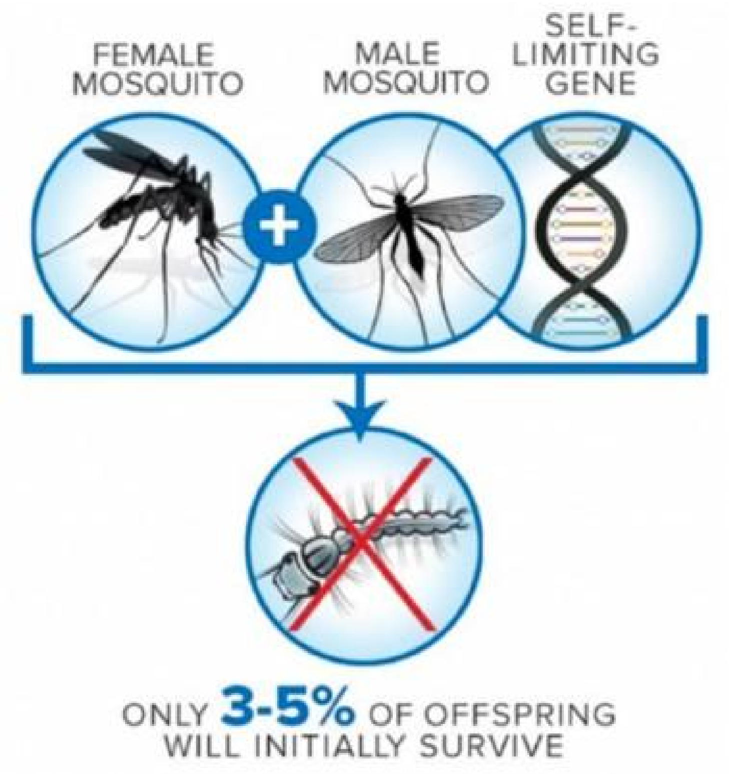

Have GM mosquitoes been tested?

Yes, Oxitec tested this in Brazil and reported that the method worked to reduce *Aedes* mosquitoes.

Have GM mosquitoes been released in Texas?

No.

Which mosquitoes does this method target?

This method has been tested targeting *Aedes* mosquitoes that can carry West Nile virus, dengue, Zika, and chikungunya.

Is it expensive to release GM mosquitoes?

This approach is still being tested and the cost of an area-wide control program using this method has not been established yet.

Are GM mosquitoes harmful to human health and/or the environment?

The FDA has not found GM mosquitoes to be dangerous and has approved a trial. In some locations local citizens have voted to prevent the use of this technology.

Release of Mosquitoes Carrying Wolbachia

This approach involves releasing male mosquitoes containing a bacteria called Wolbachia. When these mosquitoes mate with wild mosquitoes, the bacteria spreads through the mosquito population. Wolbachia is not harmful to humans. Mosquitoes carrying this bacteria can't transmit diseases.

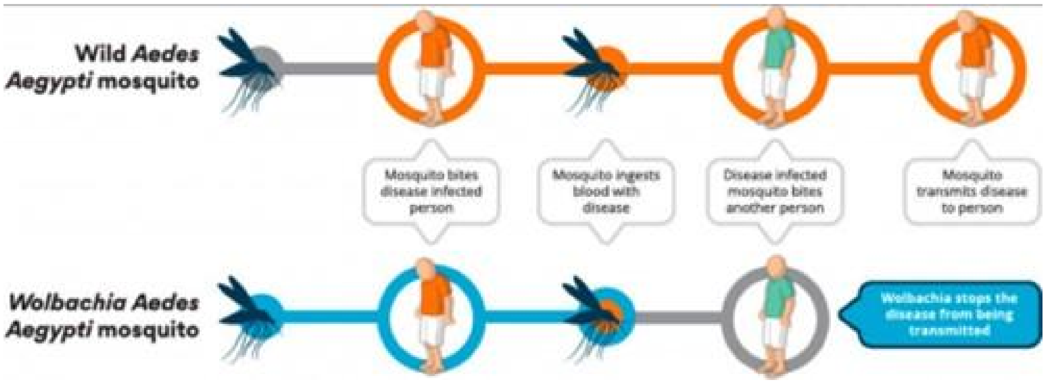

Have mosquitoes carrying Wolbachia been tested?

Yes.

Have mosquitoes carrying Wolbachia been released in Texas?

A trial is being conducted in Harris County, TX during the summer of 2019.

Which mosquitoes does this method target?

This method has been tested targeting *Aedes* mosquitoes that can carry West Nile virus, dengue, Zika, and chikungunya.

Is it expensive to release mosquitoes carrying Wolbachia?

This approach is still being tested and the cost of an area-wide control program using this method has not been established yet.

Are mosquitoes carrying Wolbachia harmful to human health and/or the environment?

The United States Environmental Protection Agency finds this method safe and has approved it.

Rating Mosquito Control Options

Think about the types of mosquito control described previously. Suppose that voters in this area approved a county-wide mosquito control program expansion. Would you SUPPORT or OPPOSE the use of each of the following control methods as part of this control program?

	Strongly Oppose	Oppose	Neutral / No Opinion	Support	Strongly Support
Adulticide (spraying insecticides to kill adult mosquitoes)	O	O	O	O	O
Larvicide (treating water sources to kill immature mosquitoes)	O	O	O	O	O
Mosquito traps (using traps in and around homes to kill adult mosquitoes)	O	O	O	O	O
Mass release of STERILE mosquitoes (cannot reproduce when they mate with wild mosquitoes)	O	O	O	O	O

	Strongly Oppose	Oppose	Neutral / No Opinion	Support	Strongly Support
Mass release of GENETICALLY MODIFIED mosquitoes (most offspring don't survive when they mate with wild mosquitoes)	O	O	O	O	O
Mass release of WOLBACHIA mosquitoes (prevents spread of disease when they mate with wild mosquitoes)	O	O	O	O	O

Why do you support/oppose the options you chose?

Conclusion of Survey

Is there anything else you'd like to add about mosquitoes, mosquito control, or your thoughts about this survey?

Where do you think would be the most useful place to provide resources about mosquito control and disease prevention in your community?

HOA Presentations

School Visits

Health Fairs

Town Hall Meetings

Church/Place of Worship

Websites

Social Media

Other

We appreciate the time and effort you put into this survey to help us better understand community members' knowledge and opinions on mosquitoes and mosquito control in your

area. We hope to use this information to develop better mosquito control programs.

To learn more about these issues and how to prevent the spread of mosquito-borne disease, you can check out the following websites:

Centers for Disease Control:

https://www.cdc.gov/features/stopmosquitoes/index.html

https://www.cdc.gov/niosh/topics/outdoor/mosquito-borne/default.html

Hidalgo County:

http://www.hchd.org/160/Vector-Control

To learn more about these issues and how to prevent the spread of mosquito-borne disease, you can check out the following websites:

Centers for Disease Control:

https://www.cdc.gov/features/stopmosquitoes/index.html

https://www.cdc.gov/niosh/topics/outdoor/mosquito-borne/default.html

Tarrant County:

http://access.tarrantcounty.com/en/public-health/disease-control---prevention.html

To learn more about these issues and how to prevent the spread of mosquito-borne disease, you can check out the following websites:

Centers for Disease Control:

https://www.cdc.gov/features/stopmosquitoes/index.html

https://www.cdc.gov/niosh/topics/outdoor/mosquito-borne/default.html

Harris County:

http://publichealth.harriscountytx.gov/About/Organization-Offices/Mosquito-and-Vector-Control

Powered by Qualtrics
